# (-)-Phenserine Attenuates Soman-Induced Neuropathology

**DOI:** 10.1371/journal.pone.0099818

**Published:** 2014-06-23

**Authors:** Jun Chen, Hongna Pan, Cynthia Chen, Wei Wu, Kevin Iskandar, Jeffrey He, Tetsade Piermartiri, David M. Jacobowitz, Qian-Sheng Yu, John H. McDonough, Nigel H. Greig, Ann M. Marini

**Affiliations:** 1 Neurology Department, Uniformed Services University of Health Sciences, Bethesda, Maryland, United States of America; 2 Department of Anatomy, Physiology and Genetics, Uniformed Services University of the Health Sciences, Bethesda, Maryland, United States of America; 3 Drug Design & Development Section, Translational Gerontology Branch, Intramural Research Program, National Institute on Aging, National Institutes of Health, Baltimore, Maryland, United States of America; 4 Pharmacology Branch, Research Division, US Army Medical Research Institute of Chemical Defense, Aberdeen Proving Ground, Maryland, United States of America; Weizmann Institute of Science, Israel

## Abstract

Organophosphorus (OP) nerve agents are deadly chemical weapons that pose an alarming threat to military and civilian populations. The irreversible inhibition of the critical cholinergic degradative enzyme acetylcholinesterase (AChE) by OP nerve agents leads to cholinergic crisis. Resulting excessive synaptic acetylcholine levels leads to status epilepticus that, in turn, results in brain damage. Current countermeasures are only modestly effective in protecting against OP-induced brain damage, supporting interest for evaluation of new ones. (-)-Phenserine is a reversible AChE inhibitor possessing neuroprotective and amyloid precursor protein lowering actions that reached Phase III clinical trials for Alzheimer's Disease where it exhibited a wide safety margin. This compound preferentially enters the CNS and has potential to impede soman binding to the active site of AChE to, thereby, serve in a protective capacity. Herein, we demonstrate that (-)-phenserine protects neurons against soman-induced neuronal cell death in rats when administered either as a pretreatment or post-treatment paradigm, improves motoric movement in soman-exposed animals and reduces mortality when given as a pretreatment. Gene expression analysis, undertaken to elucidate mechanism, showed that (-)-phenserine pretreatment increased select neuroprotective genes and reversed a Homer1expression elevation induced by soman exposure. These studies suggest that (-)-phenserine warrants further evaluation as an OP nerve agent protective strategy.

## Introduction

Organophosphorus (OP) nerve agents, such as soman, sarin, cyclosarin, tabun and VX, represent some of the most potent toxins known to man. Disturbingly, some remain stockpiled within unstable countries, making a terrorist attack on US soil or to our military overseas a potential threat. Exposure to nerve agents can induce a loss of consciousness, convulsions, paralysis, hypersecretions, diarrhea, seizures, respiratory failure and death [1], [2]. Their relatively inexpensive bulk synthesis, ease of deployment and rapid onset of symptoms to inflict mass casualties make them ideal chemical weapons. During the last thirty years, nerve agents have been used in the Iraq-Iran war in the 1980s, in the 1995 terrorist attack of the Tokyo subway and recently in the suburbs of Damascus, Syria, resulting massive casualty [3], http://www.theguardian.com/world/2013/sep/16/un-inspectors-syria-sarin-gas. OP nerve agents often provoke cognitive and neuropsychiatric adverse effects in survivors [4]–[6]. Even after 7 years, some victims of the Tokyo subway attack presented with significant declines in psychomotor and memory functions [7], signifying long-term cognitive impairment.

The primary mechanism underpinning OP toxicity is irreversible inhibition of the key cholinergic enzyme acetylcholinesterase (AChE) [1], critical in hydrolysing acetylcholine (ACh). Sudden substantial loss of AChE leads to abnormal accumulation of ACh within cholinergic synapses, resulting in the excessive stimulation of muscarinic and nicotinic receptors within the central and peripheral nervous systems [2]. In the brain, excessive stimulation of cholinergic neurons induces the release of glutamate, the overactivation of the N-methyl-D-aspartate (NMDA) receptor, and excessive influx of calcium leading to excitotoxic neuronal cell death [8]–[10]. Among OP nerve agents, soman has a median lethal concentration of 70 mg·min/m^3^ in humans within 10 min of inhalation, being more toxic and more persistent than either sarin or tabun. [11]–[14].

Currently available OP nerve agent countermeasures are aimed at reducing their peripheral and central nervous system induced actions. These include (i) atropine methyl nitrate, a muscarinic cholinergic antagonist that blocks peripheral side effects [15], (ii) HI-6, an oxime to help reactivate AChE, and (iii) the anticonvulsant diazepam, used to attenuate OP nerve agent-induced status epilepticus. Although diazepam attenuates seizures, it does not prevent neuronal injury and long-term neurological consequences [2], [9], [16]. Whereas these OP countermeasures are valuable, improvements are clearly warranted as terrorist threats remain significant.

(-)-Phenserine is a reversible AChE inhibitor structurally related to the chemical scaffold of (-)-physostigmine, which together with other reversible anticholinesterases, including huperzine [17], carbamates [18] and galantamine [19], have demonstrated anti-nerve agent activity [20]. The fundamental mechanism likely underpinning this is that pretreatment with a reversible cholinesterase inhibitor provides temporary enzyme binding to shield the active site during soman exposure when irreversible binding and inhibition would ensue. Further to providing such potential action, (-)-phenserine has several additional prospective advantages. Its lipophilic nature (log octanol/water partition coefficient value 2.2) supports a high brain delivery, with a brain/plasma concentration ratio of approximately 10∶1, as compared to unity for (-)-physostigmine [21]–[23]. (-)-Phenserine relatively selectively inhibits AChE but, unlike many clinical anticholinesterases, does so without inducing changes in protein expression [24]. It has little effect on butyrylcholinesterase (BChE) or other classical brain receptors and transporters, but has non-cholinergically-mediated post-transcriptional actions at the level of the 5′-untranslated region of the Alzheimer's and Parkinson's disease proteins, amyloid precursor protein and α-synuclein, to lower their synthesis [25]–[27]. It, together with its cholinergically inert enantiomer Posiphen [28], has demonstrated neuroprotective actions in cultured neurons [29]. Additionally, (-)-phenserine is dramatically less acutely toxic than is (-)-physostigmine in both animals and humans [22], [23], [30], and is well tolerated chronically, as assessed in Phase 2 [31] and 3 clinical trials [32] for the treatment of Alzheimer's disease.

In this study, we evaluated the neuroprotective function of (-)-phenserine against soman-induced neuropathology. As expected, pre-treatment of (-)-phenserine increased the survival rate of rats challenged with soman exposure; additionally improving movement. More importantly, (-)-phenserine significantly reduced soman-induced neuronal death. Moreover, 30 min (-)-phenserine post-treatment improved neuronal survival at a lower but significant level, whereas this dosing schedule did not impact survival rate or movement recovery, suggesting that (-)-phenserine provides neuronal protective functions independent from its AChE binding [29].

## Methods

### Animals

Experiments were performed using male Sprague-Dawley rats weighing 250–275 g (Taconic Farms, Germantown, NY). All animals were maintained on a 12 hr light/dark cycle and supplied with food and water ad libitum. The animals were acclimated for at least a week prior to surgery and a further week before drug treatment. All efforts were made to minimize the number of animals used and their suffering. The experimental protocol was approved by the Animal Care and Use Committees at the Uniformed Services University of the Health Sciences and United States Army Medical Research Institute of Chemical Defense, and all procedures were conducted in accordance with the principles stated in the Guide for the Care and Use of Laboratory Animals and the Animal Welfare Act of 1966 (P.L. 89–544), as amended. All efforts were made to minimize the number of animals.

### Placement of external jugular catheter

External jugular catheters were placed exactly as described previously [33]. Briefly, a 1.5 to 2 cm long incision was made in the ventrolateral aspect of the neck, parallel and approximately 0.5 cm lateral to midline. The external jugular vein was dissected free of surrounding tissue and stabilized with a proximally placed tie of 4-0 suture. A sterile catheter introducer was used to guide the placement of the jugular catheter. A 4-0 ligature was placed at the distal end of the catheter to secure it in place. Sterile catheters were tested for patency by flushing with sterile saline. The catheter was tunneled subcutaneously to exit via an approximately 2.5 cm incision in the interscapular region. Wound clips and/or suture material were used to close the interscapular area. Sterile heparin-saline was injected using a standardized schedule to prevent clot formation within the catheter.

### Drug treatments

#### Phenserine

(-)-Phenserine was synthesized as its water-soluble tartrate salt to in excess of 99.5% purity (21, 34) and freshly prepared on the day of experimentation. It was dissolved in saline (NaCl 0.9%) to reach a final concentration of 1 mg/ml. The dose of (-)-phenserine used in this study was 1 mg/kg intravenously (i.v.).

#### Posiphen

[(+)-phenserine] was prepared as its tartrate salt (>99.5% purity) [34], and was freshly prepared on the day of experimentation. Posiphen was dissolved in saline (NaCl 0.9%) to reach a final concentration of 1 mg/ml. The dose of posiphen used in this study was 1 mg/kg intravenously.

#### Soman

Male Sprague-Dawley rats (270–300 g) receiving nerve agent were injected with the oxime HI-6 (125 mg/kg, intraperitoneally [ip]), a cholinesterase reactivator [35], followed by soman (180 µg/kg, subcutaneously [sc], 1.6×LD_50_) 30 min later. This dose of soman was chosen because it reproducibly elicits seizures in 100% of the animals tested. Seizures were monitored by behavioral signs such as paw clonus accompanied by rhythmic ear flicks and occurred within 4–8 minutes after injection. These behaviors have been linked in other studies to onset of electroencephalographic recordings of soman-induced seizures in this model [15]. Atropine methyl nitrate (2 mg/kg, intramuscular [im]) was administered 1 min after soman. Rats were allowed to seize for 40 min and then treated with diazepam (10 mg/kg, im) to stop/attenuate the seizures.

#### Pre- and post-treatment procedures

In the pretreatment experiments, (-)-phenserine (1 mg/kg), posiphen (1 mg/kg), or saline was injected intravenously 4 hr or 30 min prior to soman administration. In the post-treatment studies (-)-phenserine (1 mg/kg), posiphen (1 mg/kg), or saline was injected intravenously 5 or 30 min after soman injection.

### Analysis of soman-induced motoric impairment

After soman exposure, animals were checked at 15 and 30 min time points to record their seizure and movement scores. Seizures were scored according to the following protocol: 0 = no seizures; 1 = fasciculations; 2 = tremors; 3 = convulsions. For movement, 0 = normal; 1 = mildly uncoordinated; 2 = impaired movement; 3 = prostrate.

#### Histological procedures

Twenty-four hours after soman or saline injection, all animals were deeply anesthetized with pentobarbital (50 mg/kg, ip) and transcardially perfused with phosphate-buffered saline (PBS, 100 ml) followed by 4% paraformaldehyde (250 ml) in PBS. The brains were removed and post-fixed overnight at 4°C, transferred to a solution of 30% sucrose in PBS for 72 hr and frozen with dry ice prior to storage at −80°C until sectioning. Coronal brain sections (40 µm) were made by a cryostat (Leica Microsystems, Bannockburn, IL), collected in 0.1 M neutral phosphate buffer, and mounted on 2% gelatin coated slides and then air dried on a slide warmer at 50°C for at least 30 min. For each brain studied, two sections were placed on gelatin-coated slides, and 14–17 slides (randomly chosen) per rat were stained with fluorojade C or cresyl violet.

#### Fluorojade C staining

Fluorojade C (Histo-Chem, Jefferson, AK) was used to identify degenerating neurons in the piriform cortex, cingulate cortex, basolateral amygdala, and hippocampus. The staining was performed in accordance with the manufacturer's instructions and as described previously [33]. Briefly, slides containing formalin-fixed brain sections were transferred to a solution of 0.06% potassium permanganate for 10 min on a shaker table to ensure consistent background suppression between sections. The slides were rinsed in deionized water for 2 min and were added to the fluorojade C staining solution for 30 min in the dark. The slides were rinsed for 1 min in each of three deionized water washes and were placed on a slide warmer rapidly, set at approximately 50°C, until they were fully dry (about 5–10 min). The dry slides were cleared by immersion in xylene for at least 1 min before coverslipping with DPX (Sigma– Aldrich, St. Louis, MO), a non-aqueous non-fluorescent plastic mounting media.

#### Determination of percent neuronal cell death

Three representative fields from each brain region per animal (n = 18/brain region) were digitally captured using a 300 color CCD camera (AxioCam, Carl Zeiss International, Thornwood NJ) attached to an Axiovert 200 microscope equipped with epi-fluorescence using an excitation wavelength of 490 and emission wavelength of 525 (Carl Zeiss International, Thornwood, NJ). Degenerating neurons were quantified by counting the number of fluorescein-positive neurons in the cingulate cortex, basolateral amygdala, hippocampus (CA1 and CA3) and piriform cortex. Data are expressed as mean ± SEM.

The percent neuronal cell death was calculated using the following formula:
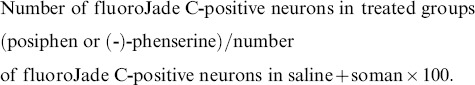



#### Quantitative real-time PCR

Piriform cortex was dissected from fresh brain exactly as described previously [36] three hours after soman treatment. The tissue was immediately frozen using a dry ice/ethanol bath and stored in −80°C freezer until use. Isolation of mRNA was performed as described previously [37]. Briefly, tissue from each brain region was lysed by adding 0.5 to 1 ml RNA STAT-60 as per manufacturer instructions (Qiagen, Germantown, MD) in a 2 ml Dounce homogenizer (35 strokes using pestle A) on ice. The lysates were transferred to sterile Eppendorf tubes and allowed to stand at room temperature for 5 min. The samples were placed at −80°C for 1 hr followed by addition of 0.1 ml chloroform per 1 ml of RNA-STAT-60, and the samples were shaken vigorously for 15 sec and allowed to stay at room temperature for 2–3 min. The cell lysates were centrifuged at 12,000×g for 15 min at 4°C. The upper aqueous phase was placed in a fresh Eppendorf tube and absolute ethanol added such that the final volume was 53% ethanol v/v. Each sample was placed on an RNeasy spin column (Qiagen, Germantown, MD) and centrifuged for 15 sec at 8000×g. The column was washed first with 700 µl Buffer RW1 then twice of 500 µl Buffer RPE, and spun for 1 min at 8000×g. RNase-free water (30–50 µl) was added directly to the spin column membrane and centrifuged for 1 min at 8000×*g* to elute the RNA. The purified RNA was subjected to RNase-free DNase digestion (Ambion, Austin, TX) and re-extracted prior to being stored at −20°C. The first strand cDNA synthesis was prepared using Cloned AMV First –strand cDNA Synthesis kit (Invitrogen, Carlsbad, CA) using 90–120 ng of total RNA for each reaction. Levels of specific mRNAs were determined using real-time fluorescence 5′-nuclease (TaqMan) assays. Reaction Mix: 12.5 µl 2× MasterMix (TaqMan Universal PCR Master Mix, Applied Biosystems, Foster City, CA) mix with 0.5 µl of GAPDH forward and reverse primer mix (10 µM), 1 µl of primers/probe mix, and 90–120 ng of RNA. The total reaction volume was taken up to 25 µl using DNase-free water. Levels of mRNA were quantified by real-time PCR (ABI Prism 7000) using the following conditions: 50°C ×2 min followed by 95°C for 10 min and finally 40 cycles at 95°C for 15 seconds and then 1 cycle at 60°C for 1 min. All probes and primers (Applied Biosystems, Foster City, CA) are listed in Table 1.

**Table 1:** Information on primers and probes used in the study.

Gene	Species	Product number	MCBI RefSeq	Exon Boundary	Assay Location	Amplicon Length	Assay Design
Ccl3	Rat	Rn01464736_g1	NM_013025.2	2–3	253	63	Probe spans exons
Atf3	Rat	Rn00563784_m1	NM_012912.1	3–4	515	88	Probe spans exons
Btg2	Rat	Rn00568504_m1	NM_017259.1	1–2	204	88	Probe spans exons
Gadd45g	Rat	Rn01352550_g1	NM_001077640.1	2–3	247	58	Probe spans exons
Cited2	Rat	Rn00586705_m1	NM_053698.2	1–2	223	71	Probe spans exons
Homer1	Rat	Rn00581785_m1	NM_031707.1	NA	1011	83	Probe spans exons
Abra	Rat	Rn00598518_m1	NM_175844.2	1–2	702	71	Probe spans exons
TNF	Rat	Rn01525859_g1	NM_012675.3	1–2	329	92	Probe spans exons
Calca	Rat	Rn01511353_g1	NM_001033955.1	2–3	216	129	Probe spans exons
Nos3	Rat	Rn02132634_s1	NM_021838.2	26–26	3900	117	Both primers and probemap within a single exon
Klf4	Rat	Rn00821506_g1	NM_053713.1	2–3	202	77	Probe spans exons
Nr4a1	Rat	Rn01533237_m1	NM_024388.1	2–3	980	64	Probe spans exons
Crh	Rat	Rn01462137_m1	NM_031019.1	1–2	155	112	Probe spans exons
Nfil3	Rat	Rn01434874_s1	NM_053727.2	2–2	1923	83	Both primers and probemap within a single exon
Fos	Rat	Rn00487426_g1	NM_022197.2	2–3	540	67	Probe spans exons
Dusp1	Rat	Rn00587176_g1	NM_053769.3	1–2	511	59	Probe spans exons
Crem	Rat	Rn01538528_m1	NM_017334.1	3–4	273	62	Probe spans exons

### Statistical analysis

Data analysis was performed by ANOVA, followed by Tukey's test for multiple comparisons unless otherwise specified. A probability of <5% is considered statistically significant. Sample size “n” refers to the number of animals. The number of animals used to determine animal survival was 17–20. The number of animals used for all other studies was 6/group.

### Chemicals

Soman (pinacoyl methylphosphonofluoridate), HI-6, atropine methyl nitrate and diazepam were provided by Dr. John McDonough at the United States Army Medical Research Institute of Chemical Defense (USAMRICD). (-)-Phenserine and posiphen were synthesized by the Drug Design & Development Section, Translational Gerontology Branch, Intramural Research Program, National Institute on Aging, National Institutes of Health, Baltimore, MD, USA. All other chemicals were purchased from commercial sources.

## Results

### Pretreatment with (-)-phenserine reduces soman-induced mortality

**Figure 1:**
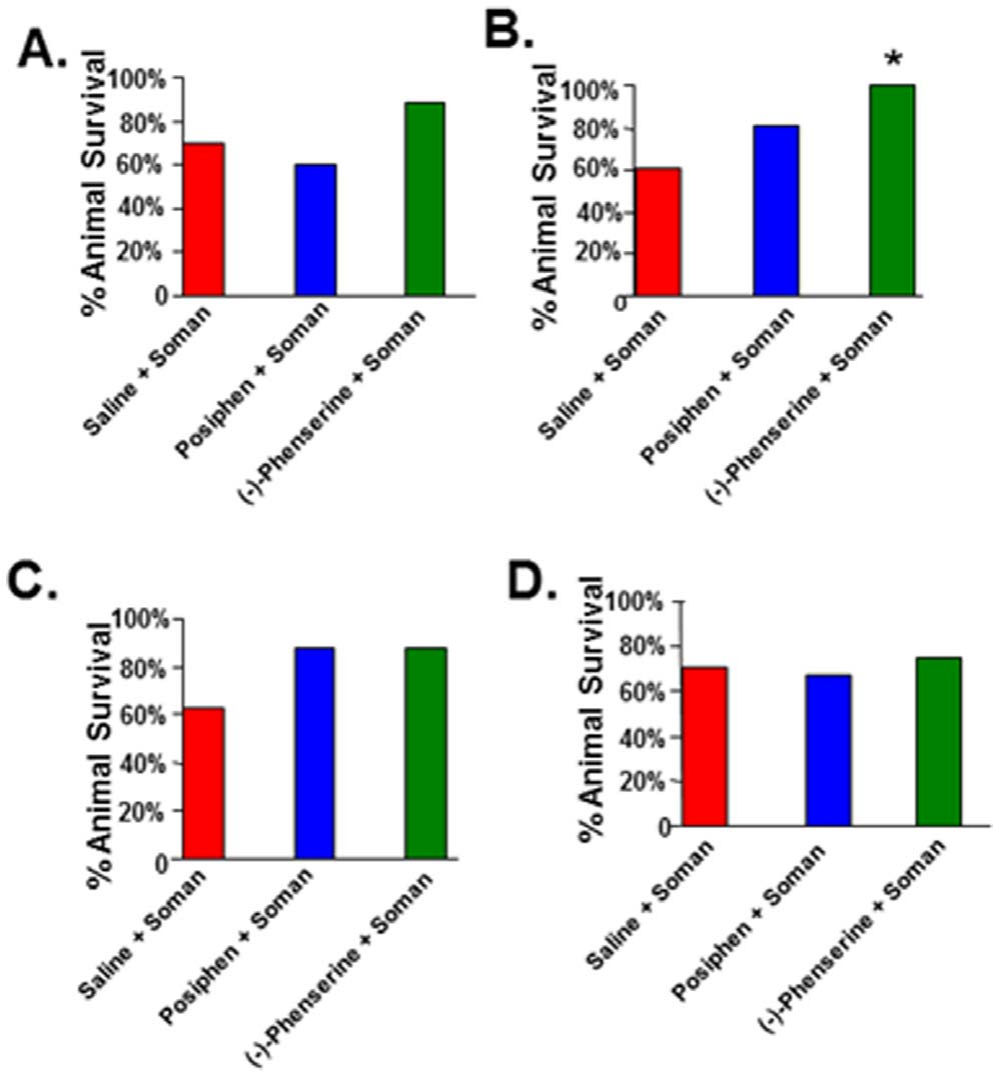
Administration of (−)-phenserine prior to but not after soman protects against soman-induced mortality. Rats were administered (−)-phenserine, posiphen or saline 4 hr (A), or 30 min (B) prior to or 5 min (C) or 30 min (D) after soman. In the 4 hr pretreatment groups of animals, 12 rats died in the posiphen group and 11 rats died in the control group. There were no deaths in the (−)-phenserine group. In the 30 min pretreatment groups of animals, 2 animals died in the (−)-phenserine group. The bar represents the percent of surviving rats 24 hr after soman exposure calculated as: number of surviving rats 24 hr after soman/total number of rats ×100. n = 17–20. *p<0.026 *vs* saline/soman by Fisher exact test.

Sprague-Dawley rats have been used as an animal model for soman toxicity studies for decades. We challenged rats with soman (180 ug/kg, sc), a dose that reliably produces status epilepticus within 10 min, and where the majority will survive for 24 hr or longer with proper care [15]. To evaluate the protective function of (-)-phenserine against nerve agent induced death, rats were administered (-)-phenserine 4 hr or 30 min before or 5 min or 30 min after soman exposure. (-)-Phenserine was administrated by intravenous bolus at a dose of 1 mg/kg, which translates to approximately 10 mg to a human (following normalization between species based on body surface area [38]. The rate of survival was calculated as the number of surviving rats one day following soman challenge (24 hr), divided by the total number of rats exposed. When (-)-phenserine was administered 4 hr prior to soman, about 90% of rats survived the soman challenge versus a survival rate of 60% and 70% for posiphen and saline, respectively. These were not statistically different from one another (Figure 1A). In contrast, administration of (-)-phenserine 30 min prior to soman provided a 100% survival rate (statistically significant *vs* saline treatment (60%) [Figure 1B]. Administration of posiphen 30 min prior to soman afforded an 80% survival rate that was not statistically different from control animals (Figure 1B). With regard to post soman treatment, a trend to improved survival was evident that did not reach statistical significance. Specifically, (-)-phenserine and posiphen provided an 88% survival, versus 63% for saline treatment when administered 5 min after soman (Figure 1C). When treatment was delayed to 30 min post-exposure, (-)-phenserine survival rate declined to 70% *vs* posiphen of 66% and saline treatment of 69% (Figure 1D).

### Administration of (-)-phenserine improves movement acutely when administered before soman exposure

Animals exposed to soman develop seizures (evaluated by their onset and severity) and peripheral nervous system dysfunction secondary to cholinergic overstimulation. Fasciculations, tremulousness as well as difficulties with ambulatory/righting ability to becoming prostrate/immobile are common features of soman exposure [39]. These signs were appraised to determine whether (-)-phenserine ameliorated the associated motor impairment induced by soman acutely. There was no significant difference in seizure onset or seizure severity associated with cholinergic overstimulation among pre- or post-treatment with (-)-phenserine or posiphen compared to the saline control animals (data not shown). For movements associated with ambulatory/righting ability, (-)-phenserine and posiphen provided positive effects following pretreatment. In the 4 h pretreatment group, 10 of 18 saline+soman rats (56%) were prostrate 15 min after the soman challenge, increasing to 12 of 18 (67%) of saline+soman rats at 30 min post soman exposure. Administration of (-)-phenserine 4 h prior to soman maintained the number of prostrated rats (6 of 17 [35%]) at 15 min and 30 min post soman. Administration of posiphen 4 h prior to soman shifted animals from being prostrate to being motor impaired. Thus, posiphen significantly lowered the number of prostrated rats induced by soman to 2 of 13 (15%) and increased the number of animals that exhibited impaired movement (11 of 13; 85%) at 15 min [*p<0.05 *vs* saline+soman animals, Fisher exact test)] {Table 2}. When re-examined at 30 min post soman, significance was not achieved in either the (-)-phenserine or posiphen group *vs* saline+soman group (Table 2). In the case of posiphen, it is possible that significance was not achieved due to the death of 5 animals. Animals that were administered saline by intravenous injection 30 min prior to soman were principally movement impaired (24 of 35 [69%]) whereas 11 of 35 (31%) were prostrate at 15 min post-soman exposure with more animals becoming prostrate (17 of 27 [63%]) at 30 min. There were no animals that were mildly uncoordinated 15 min after soman exposure in the animals that were administered saline 30 min prior to soman. Administration of (-)-phenserine or posiphen 30 min prior to soman resulted in a lower number of prostrate animals that was not statistically significant compared to saline+soman animals when examined 15 min after soman exposure; no significance was achieved in the other two categories either. However, 30 min after soman exposure, there was a significant increase in the number of animals that exhibited mild incoordination and impaired movement and a markedly reduced number of animals that were prostrate in the group pretreated with (-)-phenserine compared with animals that received saline 30 min prior to soman (**p<0.01 *vs* saline+soman). Thus, 6 of 33 (18%) animals exhibited mild uncoordinated movement compared with no animals exhibiting mild uncoordinated movement in the saline+soman group, 19 of 33 (58%) of animals exhibited impaired movement and only 8 of 33 (24%) of animals were prostrate in the (-)-phenserine group compared with 0 of 27 animals, 10 of 27 (37%) and 17 of 27 (63%) animals that exhibited mild incoordination, impaired movement and those that were prostrate in the saline+soman group of animals respectively (**p<0.001, Fisher exact test) [Table 2]. Similar results were observed when posiphen was administered 30 min prior to soman. There was a significant shift away from prostration induced by soman in the groups of animals that received posiphen prior to soman. Thus, 4 of 20 (20%) of the animals pretreated with posiphen exhibited mildly uncoordinated movement, 13 of 20 (65%) animals exhibited impaired movement and only 3 of 20 (15%) animals were prostrate compared with saline+soman groups (**p<0.01 by Fisher exact test) [Table 2]. Taken together, these results demonstrate that pretreatment with either (-)-phenserine or posiphen improves movement 30 min after a highly toxic dose of soman. When treatment with (-)-phenserine or posiphen was initiated after soman exposure, there was no statistically significant difference in motor impairment or seizure onset between groups (data not shown).

**Table 2:** Effect of pretreatment time of (−)-phenserine on movement after soman.

15 minutes after soman					30 minutes after soman					
Time prior to soman (n)	Treatment (n)	Seizure onset (n)	Mild in-coor-dination (n)	Impaired Movement (n)	Prostrate (n)	Total (n)	Mild in-coor-dination (n)	Impaired Movement (n)	Prostrate (n)	Total (n)
4 h	(−)-phenserine	4.2±1.43	1	10	6	17	0	11	6	17
4 h	posiphen	4.5±2.38	0	11*	2*	13	0	6	2	8
4 h	saline	5.6±1.52	0	8	10	18	0	6	12	18
30 min	(−)-phenserine	3.6±0.75	0	27	8	35	6**	19**	8**	33
30 min	posiphen	3.3±1.32	1	13	6	20	4**	13**	3**	20
30 min	saline	3.4±0.56	0	24	11	35	0	10	17	27

*p<0.05.

**p<0.01.

### Neuroprotective efficacy of (-)-phenserine against soman

**Figure 2:**
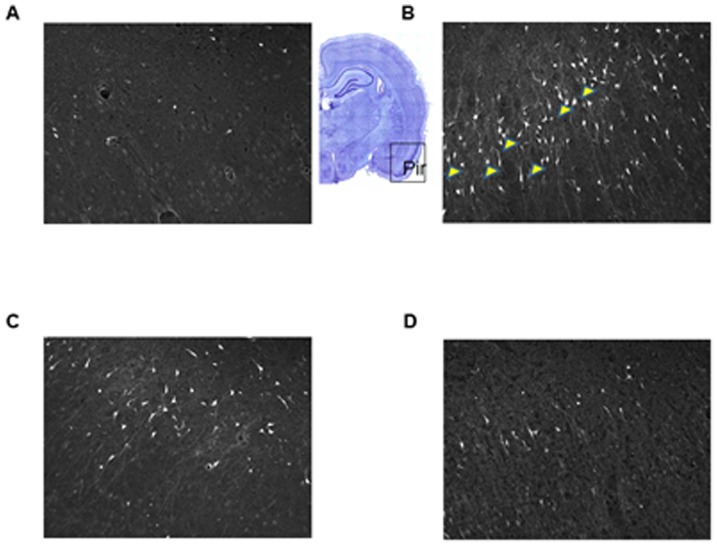
Administration with (−)-phenserine but not posiphen thirty minutes prior to soman exposure reduces neuronal cell death in the vulnerable piriform cortex. Representative photomicrographs of the piriform cortex stained with Fluorojade C staining (A–D, magnification 200×). Animals were injected with saline (A), a single dose of posiphen (1 mg/kg iv) [C] or (−)-pheserine (1 mg/kg iv) [D] thirty min prior to injection of soman (B). Animals were euthanized 24 hours after soman exposure. The piriform cortex (Pir) is outlined in the coronal section. Fluorojade C-positive degenerating neurons are indicated by the arrowheads. There was no statistically significant difference between groups of animals injected with saline and administration of either posiphen or (−)-phenserine in the absence of soman and are not shown.

**Figure 3:**
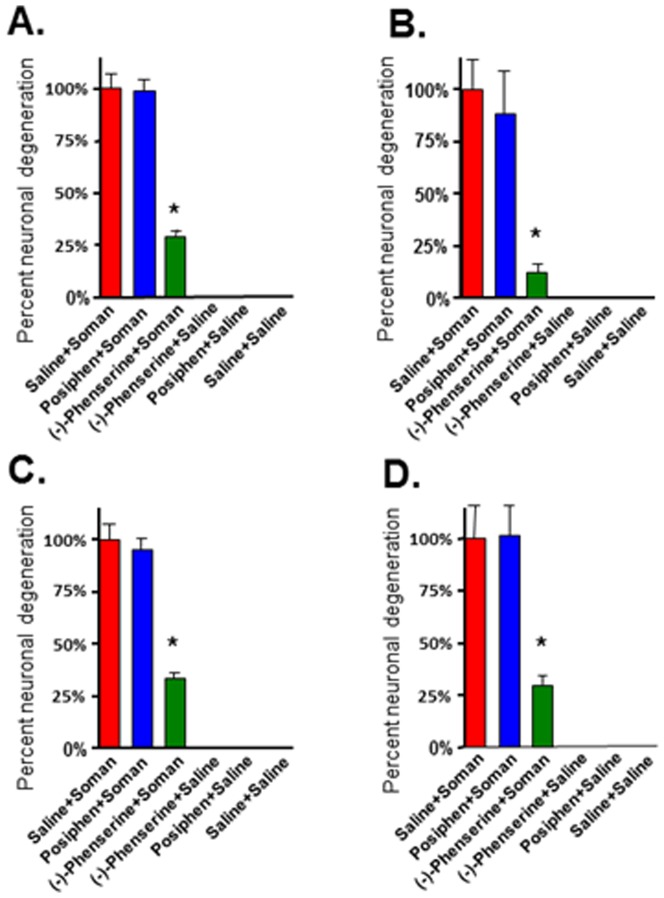
Administration of (−)-phenserine 4 hr prior to soman protects against soman-induced neuronal cell death. Rats were pre-treated with (−)-phenserine, posiphen or saline 4 hr prior to soman. Photographs were acquired from three representative fields in each brain region/animal. The bar represents the average percent neuronal cell death ± SD in the pirform cortex (A), hippocampus (B), basolateral amygdala (C), cingulate cortex (D). The number of fluorescein-positive neurons was counted by an investigator that was blinded to the treatment. n = 6/group. *p<0.001 *vs* saline/soman by ANOVA+Tukey *post hoc* analysis.

**Figure 4:**
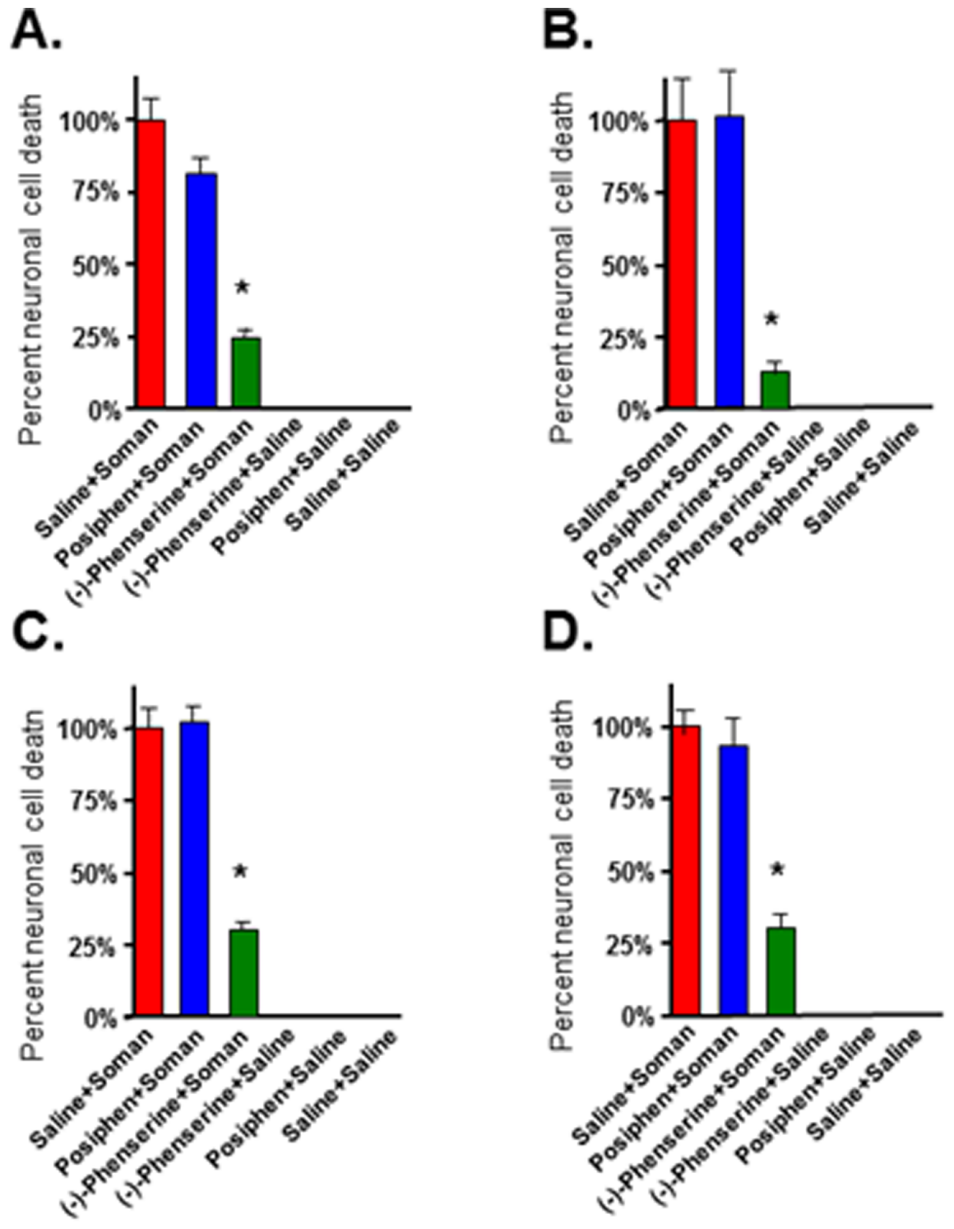
Administration of (−)-phenserine injected intravenously 30 min prior to soman protects neurons against soman-induced neuronal cell death. Rats were pretreated with (−)-phenserine, posiphen or saline, as indicated 30 min prior to soman. Slides from each brain region were randomly selected. Photographs were acquired from three representative fields per brain region per animal. The number of fluorescein-positive neurons was counted by an investigator that was blinded to the treatment. The bar represents the average percent neuronal cell death ± SD in the pirform cortex (A), hippocampus (B), basolateral amygdala (C), cingulate cortex (D). n = 6/group. *p<0.001 *vs* saline/soman by ANOVA+Tukey post hoc analysis.

**Figure 5:**
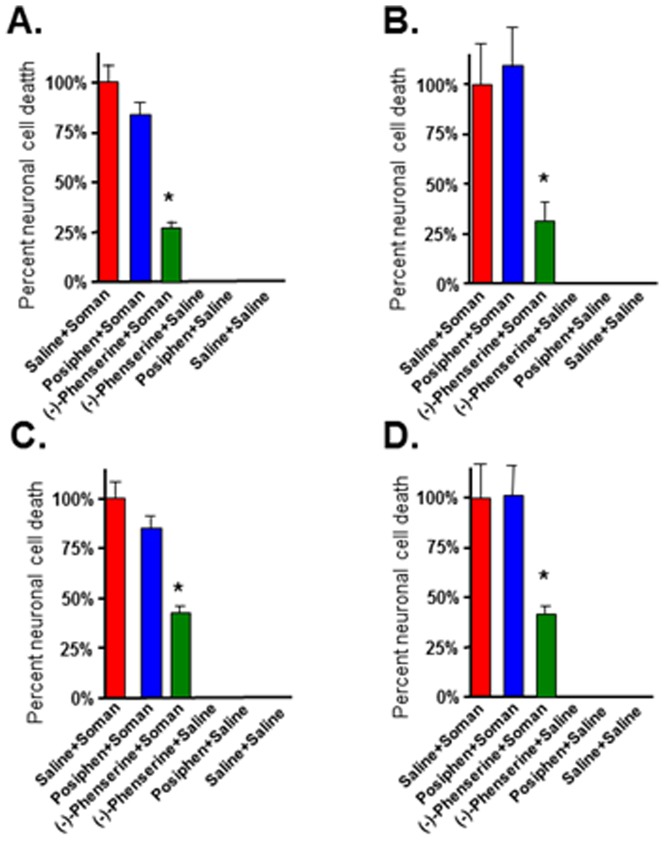
Administration of (−)-phenserine injected intravenously 5 min after soman protects neurons against soman-induced neuronal cell death. Rats were post-treated with (−)-phenserine, posiphen, or saline five minutes after soman. Three representative images were acquired from each of the four brain regions per animal. The number of fluorescein-positive neurons was counted by an investigated that was blinded to the treatment. The bar represents the average percent neuronal cell death ± SD in the pirform cortex (A), hippocampus (B), basolateral amygdala (C), cingulate cortex (D). n = 6/group. *p<0.001 *vs* soman/saline by ANOVA+Tukey *post hoc* analysis.

**Figure 6:**
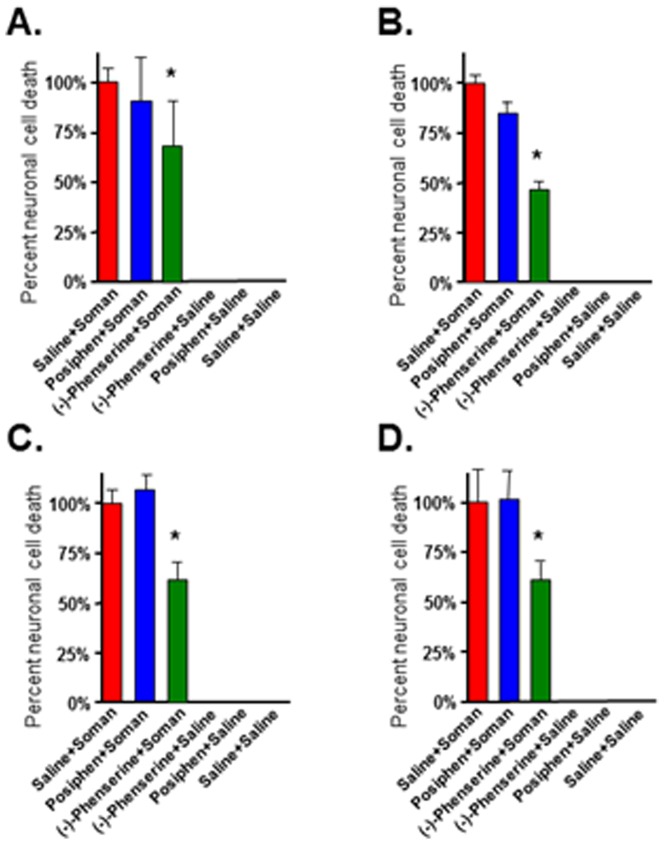
Administration of (−)-phenserine injected intravenously 30 min after soman protects neurons against soman-induced neuronal cell death. Rats were post-treated with (−)-phenserine, posiphen or saline thirty minutes after soman. Images from three representative fields were acquired for each of the four brain regions/animal. The number of fluorescein-positive neurons was counted by an investigator that was blinded to the treatment. The bar represents the average percent neuronal cell death ± SD in the pirform cortex (A), hippocampus (B), basolateral amygdala (C), cingulate cortex (D). n = 6/group. *p<0.001 *vs* soman/saline by ANOVA+Tukey *post hoc* analysis.

Neuronal cell death is a major consequence of nerve agent exposure that leads to long-term cognitive and behavioral deficits in individuals surviving initial exposure [12], [40], [41]–[45]. In the present study, fluorojade C staining was employed to evaluate phenserine-induced neuroprotection against soman-induced neuropathology, in accordance with its prior use to quantify soman-induced brain damage [33], [46]. Four regions were assessed in rat brain known to be vulnerable to soman: basolateral amygdala, piriform cortex, hippocampus, and cingulate cortex, as these brain regions display the most damage upon soman exposure [9]. The percent neuronal cell death from groups of animals treated with either (-)-phenserine or posiphen+soman was compared to saline+soman groups. The number of fluoroJadeC-positive neurons was quantified in each of the four brain regions and the percent neuronal cell death was calculated as described in Materials and Methods. Across treatment times, (-)-phenserine administration significantly protected neurons in the four vulnerable brain regions whereas posiphen treatment was not statistically different from controls (saline+soman). Representative images of the effect of phenserine on soman-induced damage in the piriform cortex are shown in Figure 2. There is a marked increase in the number of fluorescein-positive neurons (degenerating neurons) following soman (Figure 2B). In sharp contrast, administration of a single dose of (-)-phenserine 30 min prior to soman exposure strikingly reduces the number of fluorescein-positive neurons (Figure 2D) compared to saline (Figure 2A) Administration of posiphen 30 min prior to soman exposure did not significantly reduce the number of fluorescein-positive neurons in the piriform cortex (Figure 2C). Administration of (-)-phenserine 4 hr prior to soman challenge significantly reduced neuronal cell death in the piriform cortex (30.4%), hippocampus (12.5%), basolateral amygdala (33.8%), and cingulate cortex (31.2%) *vs* saline+soman [Figure 3]. Administration of (-)-phenserine 30 min prior to soman exposure significantly reduced neuronal cell death in the piriform cortex (24.7%), hippocampus (12.2%), basolateral amygdala (30.6%), and cingulate cortex (30.4%) [Figure 4]. When rats were treated 5 min after soman exposure, (-)-phenserine continued to provide neuroprotection; significantly reducing neuronal cell death to 27.3% in piriform cortex, 31.2% in hippocampus, 42.9% in basolateral amygdala, and 41.6% in cingulate cortex (Figure 5). This effect was diminished but, nevertheless, still statistically significant when (-)-phenserine was administered 30 min post soman; reducing neuronal cell death to 68.8% in piriform cortex, 46.2% in hippocampus, 61% in basolateral amygdala, and 61.6% in cingulate cortex (Figure 6).

### Changes in gene expression associated with (-)-phenserine-induced neuroprotection against soman

Following exposure of the brain to a nerve agent, such as soman, a host of responses are triggered that result in alterations in gene expression profiles. Such changes can be categorized into three groups: 1) changes that facilitate damage, 2) changes that counter damage and, 3), changes that have no effect. For a potentially protective strategy one might expect further changes in the expression of genes associated with endogenous protective pathways activated in response to soman challenge, as well as changes in gene expression that counter pathways associated with cellular demise. Microarray studies by Dillman and colleagues [47] have described time-dependent (1 hr to 7 days) changes in rat hippocampal gene expression profiles consequent to soman exposure; thereby, identifying key genes. Anchored on this, real-time PCR was used in the current study to evaluate changes in expression profiles of a select number of genes early after soman exposure with and without (-)-phenserine 30 min pretreatment, as this pretreatment time afforded the greatest protection based upon animal survival and neuropathology. Based on the practicality of the exposure procedure, animal transportation and isolation of brain tissue, a time of 3 h was chosen. Gene changes of greater than 7-fold were evaluated within the piriform cortex, a major brain region targeted by soman. Additionally, to further narrow down genes of potential interest, our selection was restricted to ones expressed within neurons or involved in programmed cell death, cell survival, or signal transduction pathways. This provided 17 target genes, listed in Table 3, amongst which some have reported neuroprotective functions e.g., AID genes (Atf3, Btg2, Gadd45g), Nr4a1 [48], [49], Fos [50], Cited2 [51], Dusp1 [52], [53], Klf4 [54], and Nfil3 [55], [56] whereas others are associated with neuronal apoptosis e.g. Nos3 [57], Crh [58], [59], Crem [60], Homer1 [61]. Others still (Ccl3 and TNF) are inflammatory markers [62], since inflammation is a known brain response to nerve agents. Finally, Abra (also known as Stars) and Calca expression profiles were evaluated.

**Table 3:** Administration of (−)-phenserine thirty minutes prior to soman differentially modulates gene expression in the piriform cortex three hours after soman.

Gene	Phenserine/Saline (fold change)	p value
Btg2	1.91	<0.001
Ccl3	1.85	<0.01
Atf3	1.76	<0.05
Gadd45g	1.51	<0.05
Cited2	1.46	<0.05
Homer1	0.54	<0.05
Abra	1.81	NS
TNF	1.51	NS
Calca	1.43	NS
Nos3	1.39	NS
Klf4	1.28	NS
Nr4a1	1.14	NS
Crh	1.09	NS
Nfil3	1.07	NS

NS: not significant.

All the selected genes demonstrated altered expression levels upon soman exposure. Among them, the expression of six genes was significantly (p<0.05) impacted by the 30 min (-)-phenserine pretreatment. Five of these, Btg2 (1.9-fold), Ccl3 (1.85-fold), Atf3 (1.76-fold), Gadd45g (1.51-fold), and Cited2 (1.46-fold), showed significant elevation, and one, Homer1 (0.54-fold) reduced expression to a level close to pre-soman treatment (Table 3). Four of the 6 genes are pro-neuronal-survival genes, whereas one, Ccl3, is an inflammatory marker that has been shown recently to be involved in adaptive changes [63] and one, Homer 1, has been reported to be involved in TRAIL-induced apoptosis [61].

## Discussion

In the present study, we demonstrate that the reversible AChE inhibitor (-)-phenserine is able to protect rats against soman exposure. This is in line with prior reports that anticholinesterases, such as huperzine A [64]–[66] and galantamine [67], are able to mitigate soman's toxic actions in animals. Reversible inhibitors that bind AChE have the potential to shield the enzyme from irreversible covalent binding by nerve agents. Additionally, for reversible anticholinesterases that possess a lower affinity for butyrylcholinesterase (BuChE) that is now known to co-regulate cholinergic neurotransmission [68], [69], the availability of uninhibited plasma and brain BuChE may provide a prospective sink to bind soman; thereby potentially lowering its toxicity [8]. This theoretical mechanism would require AChE binding to a reversible inhibitor prior to soman exposure to afford AChE protection; thereby making any putative anti-soman agent most effective when administrated shortly before contact. Like huperzine A, (-)-phenserine proved capable of protecting animals from soman-induced death. This proved to be time sensitive, with the 30 min pretreatment providing the most significant protection (Figure 1), in line with the hypothetical shielding of AChE from soman. Seizure onset and severity were not, however, altered by the pre- or post-treatment of (-)-phenserine. As in other studies [67], a caveat of the current study is the use of a single (-)-phenserine dose (1 mg/kg) to evaluate neuroprotective efficacy. This dose was selected based on its clinical relevance (1 mg/kg in rat is approximately equivalent to 10 mg in a 65 kg human [30], [32]), following normalization based on body surface area [38], in accord with the Food and Drug Administration guideline for estimating the maximum safe starting dose in initial clinical trials for therapeutics in adult healthy volunteers. This human 10 mg phenserine dose was well-tolerated in healthy volunteers and Alzheimer's disease subjects [30]–[32]. Clearly, lower and higher (-)-phenserine doses warrant evaluation in future studies to protect against soman-induced brain damage.

The significant movement improvement (Table 2) achieved by pretreatment with (-)-phenserine or posiphen suggests that protection from soman toxicity stems from more than AChE shielding alone, particularly since posiphen lacks anticholinesterase action, albeit specific metabolites possess some [70]. In both humans and rats, posiphen remains the primary drug species as assessed by both its maximal concentration in plasma and brain, and time-dependent area under the plasma concentration curve [28]. In contrast, (-)-phenserine undergoes relatively rapid metabolism (30) to generate its bisnor N-demethylated metabolite that, likewise, possesses AChE inhibitory action and remains the major active drug species [71]. Recent studies of posiphen and (-)-phenserine indicate that they, together with specific metabolites, possess both neurotrophic and neuroprotective actions that are mediated via the protein kinase C (PKC) and extracellular signal-regulated kinase (ERK) pathways [29], rather than through cholinergic mechanisms. In neuronal cultures, (-)-phenserine and posiphen are reported neuroprotective against glutamate excitotoxicity and oxidative stress and augment the survival of neural stem cells [29]. These actions appear to translate to animal studies, in which increased brain-derived neurotrophic factor (BDNF) levels [29] as well as elevated neurogenesis within the dentate gyrus, increased synaptogenesis and reduced inflammatory markers have been described [29], [79], [72]–[74].

The high and preferential distribution of both (-)-phenserine and posiphen into the CNS, reaching a brain/plasma ratio in the range of 10∶1 [22], [23] to 8∶1 [28], is in line for a therapeutic developed for neurological disorders. This brain uptake compares favorably to galantamine (brain/plasma ratio: 1.2 [75]) and huperzine A (brain/plasma ratio: 0.1 to 0.4 [76]), and relates to the high lipophilicity of (-)-phenserine (log P value 2.22) consequent to its tricyclic hexahydropyrrolo[2,3b]indole backbone and phenylcarbamate moiety [34]. The latter makes it more hydrophobic than (-)-physostigmine (brain/plasma ratio: 1∶1 [21] that too has been found useful in mitigating soman toxicity in preclinical models [77], [78]. Additionally, the longer yet still moderate reversible cholinesterase inhibition half-life of (-)-phenserine versus (-)-physostigmine (approximately 6 h [23] and 80 min [79], respectively) appears to be more in line with the calculated disappearance and evaporation of soman following its release [80], where levels are computed to be approximately maximal over the initial 60 min and gradually decline over 3 h [80]. In prior studies of (-)-phenserine involving a similar dose in rats, an inhibition of approximately 50% systemic AChE inhibition was achieved at 30 min [22] and the enzyme kinetics of the compound have been described [81].

In the wake of recent chemical attacks on civilians in Syria, as important to optimizing survival in those exposed is the development of antidotes to provide neuroprotection from resulting overactivation of cholinergic and glutamate systems leading to NMDA receptor-mediated excitotoxicity [82], and the development of persistent profound neuropsychiatric, neurological deficits (epitomized by cognitive and memory impairments, psychomotor performance deficits, somatic complaints, and non-specific mental and post-traumatic stress disorder) as well as structural alterations in human brain following nerve gas poisoning [5], [7], [44], [83]–[86]. (-)-Phenserine proved able to protect neurons against soman induced neuropathology in four brain regions known to be strikingly vulnerable to soman. Importantly, such protection extended to both pre- and post-treatment (Figures 3–6); albeit 30 min pretreatment proved best. To elucidate possible mechanisms underlying the phenserine-induced neuroprotection, analysis of a focused gene set was undertaken whose expression has been reported to dramatically change following soman exposure [47]. This investigation was performed in the piriform cortex, as damage within this brain region proved particularly pronounced, in line with previous studies [2], [9], [16]. Among 17 genes evaluated, the expressions of 6 were significantly impacted by (-)-phenserine treatment prior to soman exposure. These genes can be grouped into subcategories. The first, comprising btg2, atf3, and gadd45g, represent principal components of a core set of neuroprotective genes termed Activity-regulated Inhibitor of Death (AID) genes [48], [49]. The expressions of these genes are reported to be dependent on nuclear calcium signaling, are likely CREB target genes, and appear to promote neuronal survival *in vitro* and *in vivo*, particularly following seizure activity, through inhibition of death signal–induced mitochondrial permeability transition [48]. Hence their up regulation by (-)-phenserine is in accord with beneficial activity to counter soman neurotoxicity. Cited2 has been reported to be involved in the MAPK and PI3K pathway [87], is a regulating factor in their genes [49], and is involved in hypoxic signaling as a negative regulator of HIF-1α [88]. The gene for Ccl3 or human macrophage inflammatory protein 1α (MIP-1α), a member of the β-chemokine subfamily, is one of the most immediate and elevated gene responses to head injury [89]. Whereas the addition of ccl3 protein did not directly impact neuronal survival in cultured hippocampal neurons, its elevation in response to soman exposure may attract neutrophils to injured brain sites [90] to augment extravasation of degenerating cells and, thereby, promote neuronal regeneration and/or may be involved in adaptive responses to neuroinflammation [63]. Finally, Homer1 is reported to be involved in TRAIL induced apoptosis [61]. Whereas Homer1 mRNA levels are elevated by soman exposure, in line with its pro-apoptotic function, pretreatment with (-)-phenserine reduces it to basal levels. These gene changes can be considered a preliminary analysis but the results set the stage for a larger gene expression study to examine how (-)-phenserine's actions are mediated against soman poisoning in brain.

In synopsis, the present study demonstrates that acute treatment with (-)-phenserine, a centrally active AChE inhibitor that has proven well tolerated and active in AD clinical trials [31], [32], increased animal survival following soman exposure in a well characterized rodent model. In accord with recent cellular and animal studies [29], [72], (-)-phenserine mitigated neuronal cell death in surviving animals. Initial gene expression analysis suggests that this neuroprotection is mediated via multiple pathways, including non-cholinergic ones. Highlighted by the recent inappropriate exposure of civilians to nerve gas in Syria [81], effective antidotes remain an unmet and critical medical need, and support the further evaluation of (-)-phenserine in preclinical models of translational relevance.
